# A novel formulation technology for baculoviruses protects biopesticide from degradation by ultraviolet radiation

**DOI:** 10.1038/s41598-020-70293-7

**Published:** 2020-08-06

**Authors:** Kenneth Wilson, David Grzywacz, Igor Curcic, Freya Scoates, Karen Harper, Annabel Rice, Nigel Paul, Aoife Dillon

**Affiliations:** 1 Lancaster Environment Centre, Lancaster University, Lancaster, LA1 4YQ UK; 2 Department of Agriculture Health and Environment, Natural Resources Institute, University of Greenwich, Medway Campus, Central Avenue, Chatham Maritime, Kent, ME4 4TB UK; 3 Exosect Limited, Leylands Business Park, Colden Common, Winchester, SO21 1TH Hampshire UK

**Keywords:** Agroecology, Food microbiology, Pathogens

## Abstract

Biopesticides are biological pest control agents that are viewed as safer alternatives to the synthetic chemicals that dominate the global insecticide market. A major constraint on the wider adoption of biopesticides is their susceptibility to the ultraviolet (UV: 290–400 nm) radiation in sunlight, which limits their persistence and efficacy. Here, we describe a novel formulation technology for biopesticides in which the active ingredient (baculovirus) is micro-encapsulated in an ENTOSTAT wax combined with a UV absorbant (titanium dioxide, TiO_2_). Importantly, this capsule protects the sensitive viral DNA from degrading in sunlight, but dissolves in the alkaline insect gut to release the virus, which then infects and kills the pest. We show, using simulated sunlight, in both laboratory bioassays and trials on cabbage and tomato plants, that this can extend the efficacy of the biopesticide well beyond the few hours of existing virus formulations, potentially increasing the spray interval and/or reducing the need for high application rates. The new formulation has a shelf-life at 30 °C of at least 6 months, which is comparable to standard commercial biopesticides and has no phytotoxic effect on the host plants. Taken together, these findings suggest that the new formulation technology could reduce the costs and increase the efficacy of baculovirus biopesticides, with the potential to make them commercially competitive alternatives to synthetic chemicals.

## Introduction

Baculoviruses are dsDNA viruses that infect insects and have, since the 1980s, been used in crop protection as commercial biological insecticides^1,2^. Baculoviruses are seen as attractive biological control agents against insect crop pests for many reasons: they have a long and detailed history of research, so basic knowledge of their taxonomy, biology and pathogenicity is available^3^; they have an established profile of safety and environmental acceptability^4^; they are highly efficacious pathogens of some of the world’s most important crop pests, such as the various *Heliothis / Helicoverpa* species*, Spodoptera* spp. and *Plutella xylostella*^5^; and, finally, their use as biological pesticides is feasible because commercially-viable mass production systems are well advanced for many baculoviruses^6^. These factors have motivated the establishment of a growing commercial production of baculovirus insecticides in the Americas, Europe, Asia, Australasia and Africa^5,7^. Moreover, biopesticides are now seen as a major candidate for replacing the many chemical pesticides that have been, and continue to be, withdrawn from the market due to safety concerns^5,8^, and/or where the insect pests have developed resistance to conventional chemical pesticides^9^.

Baculovirus products, however, still represent only a $50–70 million per annum sector of a global biopesticides market estimated to be worth $2.8 billion dollars a year^10^. While a number of factors have been identified as restricting the adoption and expansion of the use of baculovirus biopesticides by growers^5^, a central problem over the last 40 years has been their short persistence on the crop after application, due to degradation by the ultraviolet (UV: 290–400 nm) radiation in sunlight^6,11–14^. In temperate cropping systems, the half-life of baculoviruses on crops can be just 2–10 days^15–17^. In the tropics, on unshaded crop surfaces, a half-life of 8 h or less has been reported^18^. This susceptibility to UV degradation severely limits their attraction to farmers as the need to apply to the crop at weekly intervals is more frequent than competing chemical insecticides, adding significantly to costs^5,19^. This higher cost in large part accounts for their current use being limited to the high-value horticulture sector where high produce prices can offset their cost^5,19^.

Overcoming this limited UV stability has been a major goal of baculovirus research since the 1980s^20,21^. Although attempts to develop UV-resistant baculoviruses through strain selection or genetic modification have been reported^22^, these have yet to identify improvements significant enough to support commercial adoption^6,23^. There has been some limited success in improving on-crop persistence through the use of tank-mixed adjuvants but these, while increasing the persistence to a limited extent, have failed to meet users’ need for a pest control level that matches that of chemical pesticides^5^. Only through the development of improved novel formulations can performance be enhanced sufficiently to expand their usefulness beyond current niche uses into major field crops^19,24^.

There have been many efforts to identify suitable additives to enhance the UV stability of baculovirus biopesticides^6,20^, and this work has yielded some promising results^25,26^. The diaminostilbene disulfonic acid-based optical florescent brighteners (e.g. BLANKOPHOR) that act as specific UV-absorbants, and are used as commercial sunscreens, have been a focus of research to evaluate their use with baculoviruses^27,28^. Metal oxides, including titanium dioxide and zinc dioxide, have also shown promise as UV protectants^29,30^, but field trials have generated mixed results with no conclusive benefits^16,31,32^.

An important issue for combining UV protectants with biopesticides is that at the inclusion rates proposed, combined with the high water volumes used to apply the biopesticide (up to 400–1,000 L per ha^33^), the quantities and cost of additives become very significant. The UV additive may be needed at 5–20 kg per ha, at a cost many times that of the active ingredient^16^; at these rates, the cost can then become prohibitive in most cropping systems^23^. It has been argued that for baculoviruses to be acceptable for use on broad field crops, any formulated product needed to fall below a cost of $20 US per treatment per ha in order to meet the economic constraints of growers and to be competitive with chemical pesticide alternatives^23^.

If the UV protectants, instead of being tank-mixed in solution or in suspension with the infectious baculovirus occlusion bodies (OB), could instead be formulated so as to be bound intimately to the OB (i.e. encapsulated by protectant formulation), then much lower rates of additive could be used. Such a formulation would need to comprise elements that were environmentally stable in the field for days to weeks, but readily able to release the encapsulated OB within the insect gut upon ingestion, so enabling the baculovirus to initiate infection.

There have been a number of reports using encapsulation as a means of protecting baculovirus OB against harmful UV radiation^20,25,34–38^. However, none of these technologies has been adopted commercially. This may be partly due to factors such as high cost, phytotoxicity, storage incompatibility and blocking of spray filters, as occurs with some particulate additives^5^. However, this is probably because the advantages conferred by the encapsulation have so far failed to meet adequately the goal of substantially improving UV-stability^6^. Previously, *Cydia pomonella* GV (CpGV), a baculovirus effective against codling moth pests, had been successfully microencapsulated with Titanium dioxide using the Particles from Gas Saturated Solutions (PGSS) system, giving a biologically-viable formulation with enhanced UV protection, as measured by spectral analysis^37,39^. However, information on the efficacy of this formulation on crops is not available.

This paper reports on the development and evaluation of a novel wax-encapsulation formulation for baculoviruses that substantially improves UV stability at low cost. The new formulation developed and tested here utilises the proprietary ENTOSTAT waxes. ENTOSTAT is a platform technology consisting of wax particles that can be co-formulated with a range of biological and chemical active ingredients. It has been previously successfully formulated with entomopathogenic fungi such as *Beauveria bassiana*^40^ and chemistries such as spinosad^41^. ENTOSTAT has not, however, previously been used as an encapsulating agent, and neither has it been used before with baculoviruses, which differ from these other active ingredients in entering their insect host via oral ingestion, rather than via the insect cuticle/skin. Therefore, this application has significant innovative potential for both ENTOSTAT technology and baculovirus formulations.

Preliminary work tested both representative stilbene-derived optical brighteners and metallic oxide absorbants such as titanium dioxide (TiO_2_), and while both showed promise, the TiO_2_ was selected as the most suitable to take forward for full formulation and testing. The specific ENTOSTAT waxes were selected on the basis of expected biological compatibility with baculovirus and commercial viability. Any formulation would also need to meet the other requirements of a practical biopesticide formulation and have storage stability to meet accepted standards^19,20^ and so this was also evaluated.

In this study, we used laboratory systems that can be calibrated to known sunlight regimen^30,42^. The initial trials in which virus formulations on glass slides were exposed to simulated sunlight was used to screen candidate formulations, the most promising of which was then used in trials on plants. In these plant trials, two different crops were included: tomato, as a representative of a major crop on which baculovirus biopesticides are used^43^, and cabbage, which has a waxy cuticle that could pose an adherence issue for a novel formulation based on waxy particulates^44^. All the work was carried out using *Spodoptera littoralis* nucleopolyhedrovirus (SpliNPV), as a model nucleopolyhedrovirus (NPV) and one already in use as a commercial baculovirus^5^. Its main target, the Egyptian cotton leafworm, *Spodoptera littoralis*, is a polyphagous caterpillar and so could be used in persistence trials on the two different target crops.

## Results

### Simulated sunlight slide exposure bioassays

#### 16 h simulated sunlight exposure

**Figure 1:**
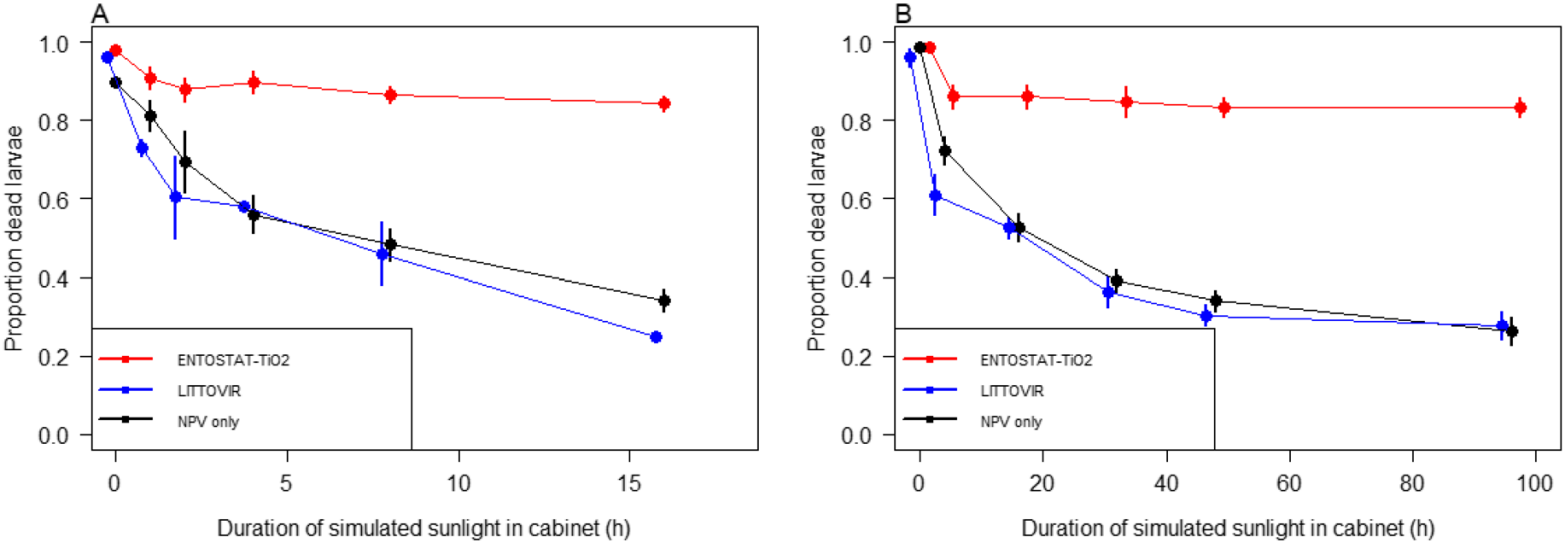
Efficacy of different NPV formulations following exposure to simulated sunlight in an ATLAS SUNTEST XLS + cabinet on glass slides for up to (**A**) 16 h and (**B**) 96 h. In both (**A**) and (**B**), three formulations were tested: non-formulated NPV (black symbols and lines), a commercial standard, LITTOVIR (blue symbols and lines), and NPV formulated in ENTOSTAT wax with Titanium dioxide, TiO_2_ additive (red symbols and lines). Symbols indicate the means and bars are ± S.E. Symbols are staggered slightly for clarity. The equivalent hourly dose of UV is 234 kJ m^−2^.

Slide bioassays showed clearly that ENTOSTAT-formulated virus with TiO_2_ additive greatly improved the stability of NPV activity when exposed to simulated sunlight in the ATLAS SUNTEST XLS + cabinet. Across the first four bioassays, when the maximum continuous exposure to simulated sunlight was 16 h, the non-encapsulated virus and the commercial virus formulation showed a non-linear decline in efficacy with increasing simulated sunlight dose, with minimum efficacy at 16 h of around 35% (Fig. 1a). In contrast, the ENTOSTAT-TiO_2_-encapsulated virus showed an initial small decline in activity but thereafter activity remained at a relatively constant level at around 87%. This observation is reflected in the statistical analysis, with a significant interaction between virus formulation and the number of hours simulated sunlight exposure (Generalised linear model, GLM: Hours exposure: χ^2^_1_ = 104.77, P < 0.0001; [Hours exposure]^2^: χ^2^_1_ = 20.96, P < 0.0001; Formulation: χ^2^_2_ = 158.19, P < 0.0001; Formulation*Hours exposure: χ^2^_2_ = 9.59, P = 0.0083). When considered alone, there was a significant decline overall in the performance of the ENTOSTAT-TiO_2_-encapsulated virus (GLM: χ^2^_1_ = 6.00, P = 0.014), but when the 0 h simulated sunlight-exposure time point was excluded, there was no significant decline in performance thereafter (χ^2^_1_ = 1.73, P = 0.19), indicating that beyond 1 h exposure to simulated sunlight there was no further degradation of virus efficacy.

#### 96 h simulated sunlight exposure

To explore this further, the duration of exposure to simulated sunlight in the ATLAS SUNTEST XLS + cabinet was increased up to a maximum of 96 h and the results again showed that TiO_2_ protects the NPV from degradation from simulated sunlight. Whilst the ENTOSTAT-formulated virus showed only a limited decline in efficacy after the first exposure to simulated sunlight and maintained > 80% efficacy throughout, the non-formulated NPV and the commercial virus showed a non-linear decline in efficacy to a minimum of around 30% (Fig. 1b). This is again reflected in the statistical analysis (GLM: Hours exposure: χ^2^_1_ = 107.29, P < 0.0001; [Hours exposure]^2^: χ^2^_1_ = 55.84, P < 0.0001; Formulation: χ^2^_2_ = 190.50, P < 0.0001; Formulation*Hours exposure: χ^2^_2_ = 9.34, P = 0.0094). When the ENTOSTAT-TiO_2_-encapsulated virus was considered alone, there was a marginally non-significant decline in performance over the 96 h exposure period (GLM: χ^2^_1_ = 3.69, P = 0.055), and when the 0 h simulated sunlight-exposure time point was excluded, there was no significant decline in performance (χ^2^_1_ = 0.31, P = 0.57), suggesting again that the virus is not subject to any further loss of efficacy after the first hour exposure to simulated sunlight.

### Simulated sunlight plant exposure bioassays

**Figure 2:**
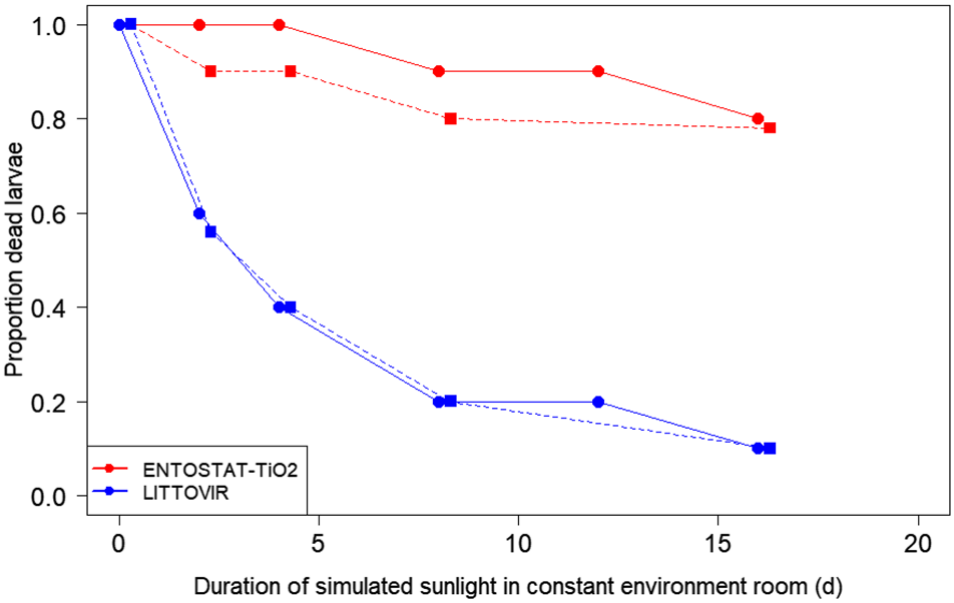
Efficacy of LITTOVIR and ENTOSTAT-TiO_2_-encapsulated NPV formulations following exposure to simulated sunlight up to 16 days in a constant environment room on tomato and cabbage plants. Cabbage = circles, solid line; Tomato = squares, dashed line. Symbols are staggered slightly for clarity. Larval mortality in the control group (dH_2_0 only) averaged 10–20% (data not shown). The equivalent daily dose of UV is 499 kJ m^−2^.

On both tomato and cabbage plants grown under 12 h light: 12 h dark simulated sunlight via LEDs and fluorescent tubes in a constant environment room, ENTOSTAT formulations incorporating TiO_2_ additive greatly increased NPV stability on plants compared to the non-encapsulated commercial standard, LITTOVIR (Fig. 2). Overall, virus-induced mortality declined non-linearly with increasing exposure to UV dose (GLM: Days simulated sunlight: χ^2^_1_ = 66.66, P < 0.0001; [Days simulated sunlight]^2^: χ^2^_1_ = 26.70, P < 0.0001) and was higher for ENTOSTAT-TiO2-encapsulated virus than for the commercial standard (Formulation: χ^2^_1_ = 179.05, P < 0.0001).

### Phytotoxicity

There was no effect of virus formulation on any aspect of the growth of the cabbage plants (Table 1).

**Table 1:** Effect of virus formulation on cabbage plant growth after 16 days.

Growth metric	Control	ENTOSTAT TiO_2_	LITTOVIR
Total number of leaves	8.167 ± 0.385	7.750 ± 0.391	8.000 ± 0.651
Number of healthy leaves	5.083 ± 0.434	4.917 ± 0.529	4.500 ± 0.701
Plant height (mm)	131.42 ± 4.39	132.75 ± 4.63	123.58 ± 6.03
Plant fresh weight (g)	27.98 ± 1.26	27.23 ± 0.87	30.24 ± 2.92
Plant dry weight (g)	2.363 ± 0.188	2.455 ± 0.098	2.388 ± 0.217

n = 12 plants per formulation. Values shown are means ± S.E. Across each growth metric, there were no significant difference between the three formulations (LM: P > 0.05).

### Titanium dioxide toxicity

**Figure 3:**
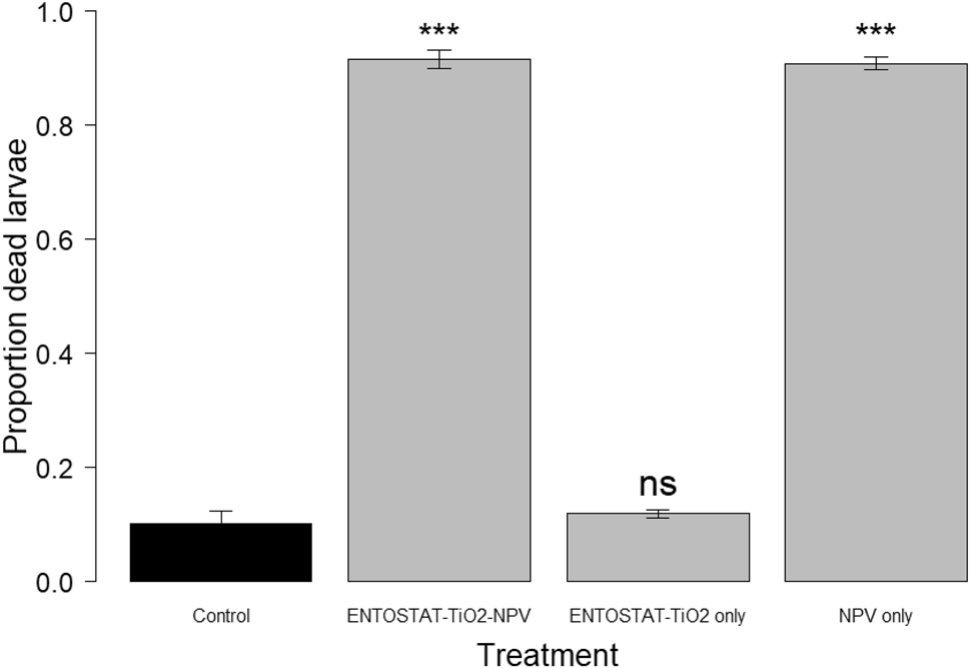
Effect of TiO_2_ and NPV on larval mortality. Bars indicate the means and error bars are ± S.E. Comparison with Control: (GLM: ***P < 0.001, ^ns^P > 0.05).

Titanium dioxide had no effect on larval mortality either when NPV-free ENTOSTAT-TiO_2_ was compared to controls that were fed dH_2_0 only (z value = 0.326, P = 0.744), or when TiO_2_-formulated NPV ENTOSTAT was compared to ENTOSTAT NPV alone (z value = 0.174, P = 0.862). Overall, however, there was a significant difference between the four treatments in the levels of larval mortality they generated (Treatment: χ^2^_3_ = 525, P < 0.0001) because the two NPV treatments caused higher mortality rates than treatments that did not include NPV (Fig. 3).

### Storage stability

After six months’ storage, virus-induced mortality was significantly lower for viruses stored at 30 °C than at 4 °C (GLM: χ^2^_1_ = 24.70, P < 0.0001) and differed across the three virus formulations (χ^2^_2_ = 29.54, P < 0.0001), with non-formulated NPV causing lower mortality in *S. littoralis* larvae than the two formulated NPVs (z value = -4.414, P < 0.0001) (Table 2). There was no significant difference between the mortality rates caused by the commercial standard and the wax-encapsulated NPV (z value = − 1.287, P = 0.198); the interaction between temperature and formulation was also non-significant (χ^2^_2_ = 0.86, P = 0.65), indicating additive effects of temperature and formulation.

**Table 2:** Virus-induced mortality of *S. littoralis* larvae inoculated with one of three SpliNPV formulations stored for 0, 3 or 6 months at 4 °C or 30 °C.

Temperature (°C)	Storage time (months)	NPV aqueous suspension n % (%)	LITTOVIR n % (%)	ENTOSTAT-encapsulated NPV n % (%)
4	0	100 (100–100)	99 (99–100)	100 (100–100)
	3	100 (100–100)	100 (100–100)	100 (100–100)
	6	93 (93–97)*	99 (99–100)	99 (99–100)
30	0	100 (100–100)	100 (100–100)	100 (100–100)
	3	100 (100–100)	99 (94–100)	100 (100–100)
	6	80 (80–90) ***	92 (92–97)	95 (95–100)

Values are means (range), n = 5 replicate bioassays. Comparison with LITTOVIR commercial standard at 4 °C (LM: *P < 0.05, ***P < 0.001).

## Discussion

Overall, the results of the work reported here using simulated sunlight show that ENTOSTAT-TiO_2_ formulated NPV is considerably more resistant to UV degradation than either non-formulated NPV or an existing commercial NPV product, and that it has storage and crop safety characteristics that are at least equal to those of existing formulations.

In any formulation of an infectious biological agent, it is important that the formulation processes result in little or no significant loss of activity^19,45^. The bioassay results confirmed that the ENTOSTAT encapsulation process, which involves heating and milling the mixture, does not reduce the infectivity of the baculovirus. Thus, the encapsulation process does not appear to damage the virus OB and, upon ingestion, the protective ENTOSTAT-TiO_2_ wax coat successfully dissolves in the insect midgut to liberate the infectious virions.

A key finding from the current study is that by combining ENTOSTAT wax with a UV protectant (TiO_2_), the efficacy of the wax-encapsulated baculovirus could be dramatically improved, even under intense UV radiation, from just a few hours to at least several days. This ENTOSTAT-TiO_2_-NPV formulation is superior to both non-formulated NPV and a commercial NPV formulation (LITTOVIR). The non-formulated virus and commercial NPV formulation had half-lives on glass slides of < 17 h continuous simulated sunlight exposure (Fig. 1b), while the ENTOSTAT-TiO_2_ formulated virus retained > 80% activity even after 96 h continuous exposure. The initial drop in the efficacy of ENTOSTAT-TiO_2_ formulated virus during the first hour of UV exposure, is most likely due to denaturing of virus that was on not fully encapsulated, whilst subsequent long-term persistence was probably due to virus that was completely encapsulated with the wax. The results from both the slide and plant systems show that the half-lives for non-formulated virus and commercial formulation fall within the reported persistence values of about 24 h in continuous simulated sunlight, and within the half-lives of 1–7 days reported in the field, depending on sunlight intensity and the degree of crop shading^2,6,20,46^. The results from the ENTOSTAT-formulated virus are very promising as no previously reported studies using a baculovirus formulation have demonstrated anything approaching this degree of UV-stability.

The slide exposure system used here (ATLAS SUNTEST XLS + cabinet), differs from many other published formulation studies in that it was calibrated to an actual sunlight exposure level, and the intensity and duration was monitored throughout using a system already validated for UV-stability studies with baculoviruses^30,35,47^. It has been pointed out that laboratory studies of baculovirus sunlight stability using ad hoc arrangements of UV or germicidal lamps not calibrated to real sunlight can be very hard to relate to field conditions^30^. The slide simulated sunlight exposure system used here also involved controlling the slide temperature, avoiding the confounding effects of the heating of glass slides, sometimes seen when slides are exposed to artificial or natural sunlight without adequate temperature control^20^. Thus, the ATLAS SUNTEST system is seen as giving a realistic model of natural sunlight under temperate conditions and has previously been used with both the bacterium *Bacillus thuringiensis* and the baculovirus *Anagrapha falcifera* multiple nucleopolyhedrovirus, MNPV^35,48^. It is widely used as an industry standard equipment for testing products for solar stability and temperature (Atlas 2018), conforming to *American Society for Testing and Materials* (ASTM) and *International Council for Harmonisation of Technical Requirements for Pharmaceuticals for Human Use* (ICH) standards for coatings, cosmetics and pharmaceutical products.

Formulations showing improved UV stability in the laboratory, however, do not always show improved persistence or efficacy in crop trials^6,35^. For example, although the UV protectant BLANKOPHOR BBH increased baculovirus persistence in lab trials, it did not increase effectiveness in field trials^49^. The crucial test of UV formulations must remain the use of plant studies, as this alone can most adequately approximate to the on-crop situation. The plant-based system used here draws on approaches that are standard in plant UV research to ensure radiation treatments were as close to the field as possible, for example filtering the UVB sources to remove wavelengths below those present in sunlight^50,51^. The crop results on tomato and cabbage show that the ENTOSTAT-TiO_2_ formulated baculovirus is substantially better than non-formulated virus or an existing commercial formulation, showing > 80% activity after 16 d diurnal simulated sunlight exposure, while the natural and commercial formulations both retained < 20% activity under these conditions. It remains to be established whether these findings are replicated in field trials under a range of natural sunlight conditions.

The bioassays reported here indicate that TiO_2_ does not, in itself, have any insecticidal activity, as the mortality of insects fed on ENTOSTAT-TiO_2_ encapsulated virus, was no greater than that of the virus alone (but possible sub-lethal effects of TiO_2_ nanoparticles have been reported in larvae of the waxworm moth, *Galleria mellonella*^52^). Photostabilized Titanium dioxide is widely used in sunscreens and cosmetic products because it has been identified as safe, a factor in its favour as a potential formulation ingredient for biopesticides^30,53^.

An issue for some formulation additives is the potential for phytotoxicity. It has been reported that when stilbene-derived UV blockers were sprayed onto plants, they had significant negative effects on the growth of several crop species tested^54^. Trials here showed that ENTOSTAT-TiO_2_ had no adverse effect on cabbage plant growth. This could be ascribed to the very low application rate of TiO_2_ when delivered as part of the ENTOSTAT formulation, which are substantially lower than in previous studies. Titanium dioxide used in suspension when sprayed at 200 L per ha would require 2 kg of TiO_2_ per ha^30^, whereas in an ENTOSTAT formulation, because the TiO_2_ is intimately bound to the wax-encapsulated OB rather than in general suspension, ≤ 20 g per ha only would be applied.

The research reported here is the first use of ENTOSTAT technology for encapsulating a biopesticide, as previous work has involved biopesticides being formulated to adhere to the outside of ENTOSTAT wax particles^40,55^. ENTOSTAT-encapsulation of baculovirus is a novel technology developed and patented during this research collaboration (see Competing Interests below for patent information). There have been previous studies using encapsulation of baculoviruses using polymers or lignin^20,35,37,39,56^. Some showed enhanced storage or UV-protection but none for the duration reported here, and only one of these studies with lignin formulation progressed to successful plant trials^56^.

An absolute requirement for a biopesticide formulation is that it can be applied through existing commercial spray systems^6^. Preliminary results, using a spray boom attached to a Hardi ilemo 1,000 L orchard sprayer using a lilac Albuz ATR80 nozzle, show that the ENTOSTAT formulation is sprayable (data not shown).

Another important issue for any biological pesticide is storage stability, as agents that cannot be formulated to remain stable for long periods have low viability as commercial biopesticides^33,45^. Baculoviruses are relatively stable robust agents^23^, but as formulated in water or glycerol have struggled to match the 2–4 years storage stability of chemicals and require freezer or cool chain storage^5,20,25^. The results presented here show that ENTOSTAT-TiO_2_-NPV has storage properties at least equal to that of a commercial glycerol-based formulation at 30 °C over 6 months. However, longer-term trials are needed to see if the ENTOSTAT-formulated NPV can reach the 18–24 month target proposed for biopesticides^20^.

A key issue with any commercial formulation is to ensure that the financial benefits outweigh the costs^19^. Whilst many studies have highlighted formulation additives that improve the performance of biological agents^20,57^, very few discuss the cost–benefit ratio of the additives/formulations. The use of UV protectants in biopesticides has been constrained because the typical inclusion rates are too high and too costly for routine field use^5,13,30^. With ENTOSTAT-TiO_2_, the UV protectant is tightly bound to the baculovirus OB, so only small quantities (g per ha) are required compared to when blockers are used in suspension (Kg per ha). For example, TiO_2_ applied as a suspension at an effective 187 L per ha was costed at $18.06 per ha, a significant issue that has constrained its adoption so far in commercial formulations^30^. In an ENTOSTAT-TiO_2_ formulation the equivalent cost for the TiO_2_ would be around $0.08 per ha (in 2003 prices). Thus ENTOSTAT-TiO_2_ can be said to address this issue as the key ingredients, wax and photo-stabilizing TiO_2_, are readily and cheaply available, and at the rates that would be used in the field would cost < $1 per ha.

A major cost associated with any commercial biopesticide is that associated with producing the active ingredient, and this is particularly true of baculoviruses, which commercially are produced in vivo. If the UV persistence of baculoviruses can be increased by using the ENTOSTAT-TiO_2_ formulation, then the amount of baculovirus applied might be drastically reduced. The current high application rates of around 1–5 × 10^12^ OB/ha are needed to overcome the short persistence issues. It has been suggested that with more effective UV protection, application rates could be reduced by a factor of × 10^30^. It is thus conceivable that adopting ENTOSTAT-TiO_2_ formulation could enable producers to reduce active ingredient rates and that this would have a drastic effect on the cost of baculovirus products. It may even enable baculovirus products to reach the goal proposed by Reid et al.^23^ of bringing biopesticide product costs below US$ 20/ha; making them, for the first time, competitive in broad acre crops. This could vastly increase their potential market share. Greater UV persistence could also allow biopesticides to be sprayed prophylactically in response to cues of imminent pest attack (e.g. large numbers of reproductive adults in traps) rather than timed precisely to coincide with pest appearance on crop. Moreover, it may be hypothesised that baculoviruses could be successfully micro-encapsulated in ENTOSTAT waxes with a broad range of different additives to give additional desirable characteristics, such as phagostimulation, broader host ranges, enhanced kill rates, etc., to deliver much ‘smarter’ biopesticides that enhance the beneficial properties of these biological entities^24^.

The issue of limited UV persistence is not restricted to baculovirus biopesticides, but also affects all of the major groups of pathogens used in crop protection^20^. Thus, it is possible that the ENTOSTAT-encapsulation system described here could be used with other pathogens, such as the widely-used bacterium *Bacillus thuringiensis*, whose on-crop persistence while longer than that of baculoviruses, is similarly limited to a few days^20,48^.

## Conclusion

We present here a novel formulation technology that effectively safeguards the efficacy of a baculovirus biopesticide by protecting the sensitive viral DNA from damaging UV radiation in sunlight on the crop by encapsulating it in an ENTOSTAT-TiO_2_ waxy coat. This technology is a highly promising candidate formulation, whose adoption in baculovirus and other biopesticide formulations could greatly increase the persistence and effectiveness of biopesticides while reducing costs. This novel formulation could be a means of greatly expanding the use of biopesticides to move them from the role of niche products for high-value and protected crops into large-scale field crop use. This would meet the need for a safer, more ecologically-acceptable, pest control approach to replace those chemical pesticides that have been, or are currently being, removed from the market-place in response to public pressure for more environmentally-sustainable crop production.

## Materials and methods

### Insects

Egyptian cotton leafworm (*Spodoptera littoralis*) used were originally collected in Egypt and had been maintained at Lancaster University since 2011. Except where stated, larvae were reared in isolation in 25 mL plastic pots containing a wheatgerm-based semi-artificial diet^58,59^.

### Virus

The *S. littoralis* nucleopolyhedrovirus (SpliNPV) used here was NRI strain #0,084 produced in the laboratories of the Natural Resources Institute, University of Greenwich, and purified for formulation using a standard purification technique for NPV^45,58^. The virus was enumerated using a Neubauer improved haemocytometer and viewed under phase contrast microscopy at × 400 using a Leica DMR microscope^45^. The virus was freeze-dried using the published protocol^58^ in a SUPERMODULOYO 20 (Edwards).

### Surface dose bioassays

The laboratory bioassays reported here employed a modified surface dose bioassay method^58,60^ that utilised 96-well flat-bottomed cell culture plates (CORNING COSTAR). The bioassay used a smooth semi-synthetic diet using ground wheatgerm^58^ and was dispensed into each well before being stored in a fridge (4 °C) for later use. For bioassays, virus formulations were prepared in dH_2_0 containing 4% food dye (Dr. Oetker). After vortexing, 10 µl of test suspension was pipetted onto the surface of the diet in the 96-well plates in a structured randomised pattern. After drying, a single starved L2 larva was added to each well and each plate was wrapped in PARAFILM. These were then left for 24 h in an incubator at 27 °C after which, each larva was transferred to an individual 25 mL plastic pot containing fresh diet. After two days, handling deaths and missing larvae were recorded and mortality assessed at 8 and 15 days post-inoculation.

### Formulation process

The formulation process is proprietary information and so only brief details are provided here. Pilot studies tested a range of different waxes, chosen based on their suitability for formulation with NPV. The NPV was incorporated into the selected ENTOSTAT wax in a melt phase with a virus loading of 2% NPV/wax (w/w) and stirred to form a uniform liquid using a high sheer blender. The UV protectant was then added, and the mixture cooled before being milled using a kibbling mill and micronized in a jet mill to a fine powder (X50 ~ 5–15 um). Photo-stabilised Titanium dioxide (TiO_2_) was selected as the formulation UV protectant. The suspended concentrate (SC) formulation was made by suspending the NPV/wax micro-powder with a blend of proprietary combinations of wetting agents, dispersers, rheology modifiers and other co-formulants with TiO_2_, to create a uniform SC. Unlike non-formulated NPV, it is not possible to visually determine the concentration of OBs in wax-formulated virus, therefore the virus loading for the ENTOSTAT-TiO2-encapsulated NPV is an estimate based on how much NPV was added into the formulation.

### Simulated sunlight slide exposure bioassays

#### Slide exposure

Test formulations were applied to blank ground glass slides. 200 µL of formulation was applied to each slide as ten 20 µL droplets then air dried at room temperature prior to exposure. The slides were exposed to UV using an ATLAS SUNTEST XLS + cabinet with the SunCool attachment comparable to other UV studies^35,47^. The chamber was set to apply 65 W m^−2^ using the daylight filter and was calibrated to match standard sunlight exposure at solar noon on the vernal equinox at Miami, Florida^61^; the chamber temperature was standardised to 20 °C using the SunCool attachment. Five slides of each formulation were exposed for each time point tested over 0–96 h. Slides were arranged within the cabinet in a randomised three by five formation, to minimise the effect of any variation in UV exposure across the cabinet. After exposure, slides were immersed in 0.02% sodium dodecyl sulphate (SDS) and brushed to re-suspend and recover the exposed formulation that was then placed into a universal vial for storage at 4 °C until bioassaying.

Seven replicate bioassays were completed across eighteen blocks in a structured randomised design. Three formulations were tested: non-formulated virus, a commercial standard (LITTOVIR), and NPV formulated in ENTOSTAT wax with TiO_2_ additive. The first four replicates included UV doses up to 16 h continuous exposure (0, 1, 2, 4, 8 and 16 h). The final three replicates included doses up to 96 h continuous exposure (0, 4, 16, 32, 48 and 96 h), again comprising 24 larvae per formulation per UV dose.

### Simulated sunlight plant exposure bioassays

Virus formulations were applied to plants that were then exposed to artificial lighting that included wavelengths in the UV range of the spectrum (see below). Two plant species were used: tomato (variety Ailsa Craig) and cabbage (variety Greyhound). These were grown from seed (Moles Seeds, Colchester, U.K.) in a glasshouse in John Innes No. 2 compost. When the plants were at an appropriate stage of growth (8–12 weeks old), the plants were sprayed with either dH_2_O (control), LITTOVIR (6 × 10^8^ OB/mL) or ENTOSTAT-TiO_2_-encapsulated NPV (~ 7 × 10^8^ OB/mL). Between one and three plants were used per UV dose per treatment group. The suspension was applied evenly to the plants using 1.25 L pressure sprayers (Hoselock, Birmingham, U.K.) immediately following preparation. Plants were then left to dry overnight in a glasshouse before being moved to the controlled environment (CE) room the following morning (25 ± 2 °C).

Plant exposure to simulated sunlight used facilities and approaches that we have described previously^50^. Plants were arranged in a completely randomised design in a CE room under artificial illumination provided by LED arrays (Valoya BX180, Valoya Oy, Helsinki, Finland) for photosynthetically active radiation (400–700 nm, mean irradiance over the growing area 300 micromole quanta m^-2^ s^−1^), and fluorescent tubes for UVA and UVB radiation (UVA340 and UV313 respectively, both from Q-Panel Laboratory Products, Bolton, UK). The UVB tubes were filtered with 0.13 mm thick cellulose diacetate (Clarifoil, Courtaulds Ltd, Derby, UK) to remove wavelengths below ~ 290 nm. The light environment was measured using a double monochromator scanning spectroradiometer (model SR991-v7; Macam Photometrics, Livingston, UK). The UV treatment provided a mean total UV irradiance (290–400 nm) of 11.55 W m^−2^. The LED lights were switched on for 12 h per day, which gave a daily UV dose in the plant system roughly equivalent to 2 h in the ATLAS SUNTEST system (i.e. 499 kJ m^−2^ day^−1^). The control treatment (no UV) was provided on the same bench as the UV treatments, but used UV313 UVB tubes wrapped in clear UV-opaque polyester (Lee Filters, Andover, UK) which absorbed 97% of the UV less than 400 nm (unweighted UV irradiance 0.36 W m^−2^).

Once exposed, the plants were removed from the CE room and 5–15 leaf discs were cut from each plant using a 5 mm corkborer. These were each immediately placed in an individual cell of a 25-well square plate (10 cm × 10 cm). Individual L2 *S. littoralis* larvae that had been starved for 4 h were then added to each cell of the plates and bioassayed as detailed above. Each treatment group comprised 10–15 larvae per UV dose, making a total of 120–270 larvae per bioassay.

### Phytotoxicity

To determine any effects of the ENTOSTAT-TiO2-encapsulated NPV on the growth of the plants, the following attributes were measured in the 36 cabbage plants used in the final plant bioassay prior to cutting out the leaf discs at day 16 post-application: total number of leaves, number of healthy-looking leaves, plant height, fresh weight and dry weight. Two control treatments were used for comparison: dH_2_O (negative control) and LITTOVIR.

### Titanium dioxide toxicity

To determine if there was any mortality associated with the TiO_2_ additive, L2 *S. littoralis* larvae were inoculated with one of four treatments: dH_2_O (control), ENTOSTAT-TiO_2_-encapsulated NPV, ENTOSTAT-TiO_2_ wax blank (lacking NPV), and non-encapsulated SpliNPV using the standard bioassay. Each bioassay comprised 60 larvae per treatment (12 in the controls) and was repeated four times.

### Storage stability

To compare the storage properties of three formulations of SpliNPV, they were stored at two temperatures (4 °C and 30 °C) and sampled at three time-points (0, 3 and 6 months). The three formulations were: freeze-dried SpliNPV in aqueous suspension; LITTOVIR (commercial standard SpliNPV, Andermatt Biocontrol); and ENTOSTAT-encapsulated SpliNPV. Replicate samples of the two non-commercial virus formulations were bottled in air and sealed, as per the commercial standard LITTOVIR. Five replicate bottles of each virus sample were bioassayed at each time point and storage temperature (i.e. a total of 30 bottles per bioassay), conducted over a single 5-day period, thus providing five independent replicates of each formulation at each storage temperature and time-point. Bioassays used the standard 96-well plate surface dose assay with 30 larvae per formulation. A single dose of each virus was used (5 × 10^6^ OB/mL), chosen to achieve close to 100% mortality at time point 0 (i.e. pre-storage). Larvae in the control group were exposed to an aqueous suspension of the wax blank formulation.

### Statistical analysis

Data analyses were performed using the R statistics package (R Statistical Software, version 3.3.3 2017-03-06^62^). Mortality data were analysed using logistic regression (generalized linear models, GLMs, with binomial errors and logit link function) using a stepwise deletion approach. All other analyses used linear models (LMs) and data were tested for normality and transformed if required.

## Data Availability

All data will be made available on Dryad upon acceptance.
